# Could Animal-Assisted Therapy Help to Reduce Coercive Treatment in Psychiatry?

**DOI:** 10.3389/fpsyt.2019.00794

**Published:** 2019-11-14

**Authors:** Sonja Widmayer, Stefan Borgwardt, Undine E. Lang, Christian G. Huber

**Affiliations:** Klinik für Erwachsene, Universitäre Psychiatrische Kliniken Basel, Universität Basel, Basel, Switzerland

**Keywords:** compulsory treatment, animal-assisted therapy, psychiatry, aggression, prevention

## Abstract

For psychiatric patients, compulsory admission and coercive measures can constitute distressing and sometimes traumatizing experiences. As a consequence, clinicians aim at minimizing such procedures. At the same time, they need to ensure high levels of safety for patients, staff and the public. In order to prevent compulsory measures and to favor the use of less restrictive alternatives, innovative interventions improving the management of dangerous situations are needed. Animal-assisted therapy (AAT) is being applied in a variety of diagnoses and treatment settings, and could have the potential to reduce aggression and psychopathology. Therefore, AAT might be of use in the prevention and early treatment of aggression, and might constitute a promising component of treatment alternatives to forced interventions. To our knowledge, no study evaluating the effect of AAT on compulsory measures in persons with psychiatric diseases has been published up to date. This narrative expert review including a systematic literature search examines the published literature about the use of AAT in psychiatry. Studies report reduced anxiety and aggressiveness as well as positive effects on general wellbeing, self-efficacy, quality of life and mindfulness. Although literature on the applicability of AAT as a component of preventive or de-escalating treatment settings is sparse, beneficial effects of AAT have been reported. Therefore, we encourage examining AAT as a promising new treatment approach to prevent compulsory measures.

## Introduction

Mental health care has to exert multiple functions: primarily, psychiatry has to offer treatment options to enable patients’ restitution of mental health and an optimal quality of life (1–3). However, in addition, psychiatry is also tasked with the role to protect the patients and others from dangerous situations caused by mental illness, and to provide care for patients that would normally agree with treatment, but are unable to do so due to their impaired judgment (4, 5). This makes it necessary to be able to resort to coercive measures like compulsory admission, safety measures (e.g., seclusion or fixation), and involuntary treatment, in specific situations (2, 3). For psychiatric patients, these measures can constitute distressing and sometimes traumatizing experiences (6). In addition, coercion can increase stigmatization of psychiatry and psychiatric patients (7–9). As a consequence, clinicians aim at minimizing such procedures (10–13). In order to prevent compulsory measures and to favor the use of less restrictive alternatives, innovative interventions improving the management of dangerous situations are needed.

The main indication for the use of coercion in psychiatry is to avoid danger for the patient or others, which is caused by aggressive behavior against others (i.e., aggression, violence) or the patient (i.e., self-directed aggression, self-harm, suicide attempts) (3). These risk situations can occur due to acute or chronic aggressive patient behavior with a variety of different causes and triggers (1).

In order to prevent or reduce coercive treatment in psychiatry, innovative treatment approaches and interventions are needed. These interventions could, e.g., directly target at reducing the probability of risk behavior, thus reducing the need for coercive measures (7). On the other hand, they could also aim at improving illness-related factors promoting aggressive behavior (e.g., emotion regulation, coping with stressful situations, and anxiety) (14). In addition, measures for the prevention of risk situations and, therefore, coercion in psychiatry should have a positive benefit-risk-assessment.

Animal-assisted therapy (AAT) has gained increasing interest in clinical psychiatry and could have the potential to prevent or reduce impending risk behavior and coercion (15). Currently, AAT is more and more employed in psychosocial facilities. It is being assumed that peaceful contact between humans and animals has positive effects on the wellbeing of persons with a wide variety of diseases (16). For example, there have been positive effects for people with somatic, intellectual, and mental disabilities, children with developmental problems, geriatric patients, or persons after surgery (15–19).

Cirulli et al. (16) conducted a review of the existing literature on psychiatric patients and concluded that in order to understand the underlying mechanisms that play a role in animal-human interactions, further research would be needed. Also, they pointed out the need for more standardized AAT treatment protocols. The reviewed studies indicated that animals absorb human attention in an innocent, non-threatening manner that allows persons to calm down.

A systematic review on randomized controlled trials by Kamioka et al. (20) identified 11 studies. Their quality according to Cochrane criteria was too low to perform a meta-analysis. The authors resumed that AAT could be an effective intervention in persons with psychological and behavioral difficulties. For example, persons with depression, schizophrenia, or substance use disorders could benefit from AAT—with the premise that they have a positive relation to animals.

A recent review of randomized controlled trials on AAT was performed by Maujean et al. (17). These authors also criticized the deficient quality of the studies. Eight publications could be included. Identified methodological weaknesses were—among others—a missing control group, differences in outcome variables, and missing assessment of the specificity of positive effects during AAT. It therefore remained unclear whether the positive effects might have been merely caused by the higher attention given to the patients due to the intervention instead of the intervention itself. There have been no reports of negative effects due to AAT.

The only meta-analysis on AAT was conducted by Nimer and Lundahl (21). The authors included every type of AAT and did not use any restrictions regarding the examined patient population, which lead to the inclusion of 49 studies. The authors found moderate positive associations of AAT with improvement of autism symptoms, medical difficulties in general, behavioral problems, and emotional wellbeing.

O’Haire et al. (22) systematically reviewed literature on AAT for trauma, including posttraumatic stress disorder (PTSD). They examined six studies with participants who were survivors of childhood abuse and military veterans and found reduced depressive and PTSD symptoms, and reduced anxiety. Because of a low level of methodological rigor in most studies, the authors indicate the preliminary nature of this area of investigation.

AAT is most commonly used in pediatric care and in nursing homes. It helps to decrease children’s pain, especially in pediatric palliative care (23) and is applied according to a manual, the “Therapy Animals Supporting Kids” (TASK) Program (24). In nursing homes, AAT seems to increase mental and physical activity in elderly persons. Due to these positive effects, in Switzerland, approximately 80% of the nursing homes integrate animals into their daily routines. Some uncounted number of nursing homes even allow the residents to take their personal pets into the homes, even though major hygienic challenges result.

Around 60% of psychiatric clinics house animals on their premises (18) and it has been shown that the presence of cats positively influences patient satisfaction in psychiatric wards (18).

The dog as the prototype of a companion animal (25) is the most commonly used animal in AAT (26). Researchers have suggested that dogs would reduce stress and fear in human beings. Studies including healthy participants reported positive effects of the presence of a dog on cortisol level, blood pressure, and pulse frequency (25).

In summary, aggression against others and self-directed aggression are frequent causes for compulsory measures and involuntary treatment in current psychiatric clinical practice. AAT could be an innovative approach to reduce aggression in different patient populations, but up to now, no publication has specifically examined this issue. Furthermore, there are—to the authors’ knowledge—currently no studies directly evaluating the effect of AAT on the frequency or use of coercive measures in psychiatry. Thus, the current mini-review aimed at examining the published literature on the use of AAT in psychiatry with a focus on applicability to reduce risk behavior and improve illness-related factors promoting aggressive behavior as proxies for the potential to reduce coercion in clinical psychiatry.

## Methods

As AAT in psychiatry with the aim of reducing aggression and coercion has to be considered as an emerging field, meta-analyses currently would only be of limited use. We therefore conducted a narrative expert review with a systematic literature search.

### Search and Selection Strategy

Author SW searched the PubMed and PsycINFO online databases using a combination of search terms related to AAT, psychiatry, aggression, and coercion. There was no literature specifically focusing on coercion. Therefore we focused on domains (aggression, agitation, anxiety) associated with reduction of coercion. We applied no restriction on start date until June of 2019. Reference lists of included literature were screened for additional applicable publications. SW screened all studies according to the following inclusion criteria. We included longitudinal, cross-sectional, and case-control studies (journal articles, book chapters, and dissertations) reporting the effects of animal-assisted interventions on any psychiatric symptom. We included all studies with an age of cases/controls of at least 18 years of age. We applied no language restriction and required patients to have a professionally established psychiatric diagnosis according to DSM or ICD.

### Data Extraction

We performed qualitative analysis of all included publications. The main outcome variables were the symptom severities as reported in the individual studies. We extracted population details including diagnosis, the measured symptoms, and the type of AAT that was applied.

## Results

### Literature Search

**Figure 1:**
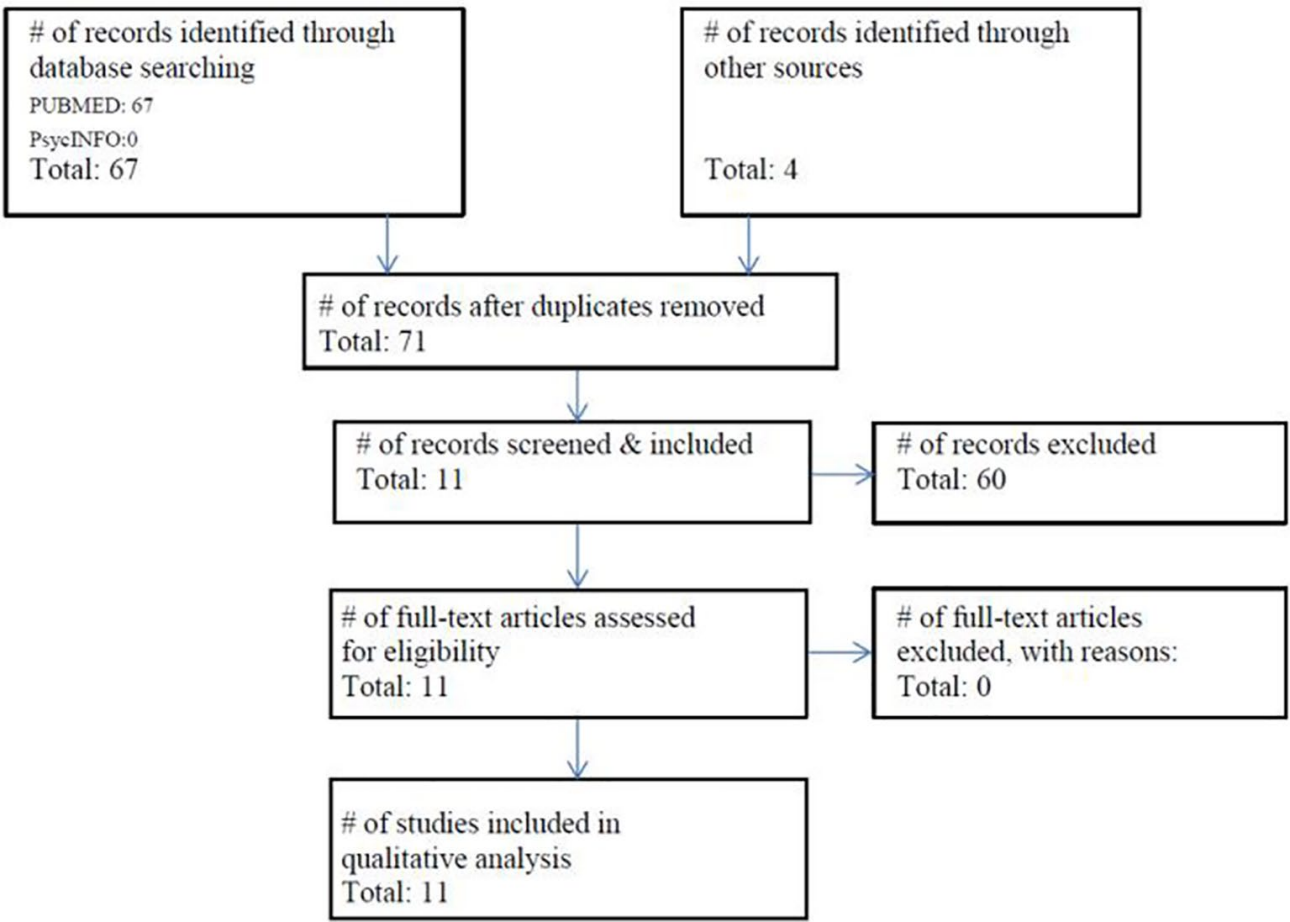
Flowchart of the literature search (21.06.2019) and included studies according to the PRISMA guidelines (27).

The literature search identified 71 possible studies of interest. After screening and applying in- and exclusion criteria, 60 studies were excluded. Using the preferred reporting items of systematic reviews and meta-analyses (PRISMA) template, we summarize the study selection procedure in **Figure 1**.

The final sample consisted of 11 studies; **Table 1** gives an overview of them showing population details, measured symptoms, and type of AAT.

**Table 1:** Overview of the effects of AAT in the included studies.

Author, Year	Population			Variable											Type of AAT					
	n patients with AAT	n patients without AAT	Diagnosis	Well-being	Anhedonia	Quality of Life	Mindful-ness	Depressiveness	Rumination	Self-Efficacy	Trauma	Verbal agitation	Anxiety/Fear	Aggression	Dog	Sheep	Cattle	Cow	Horse	Not indicated
Barker and Dawson, 1998 (26)	230	230 (same patients)	Psychosis, mood disorders										✓		✓					
Berget et al., 2008 (28)	60	30	Schizophrenia, affective disorders, anxiety, personality disorders			✓				✓						✓	✓	✓	✓	
Hediger et al., 2019 (19)	19	19 (same patients)	Acquired brain injury	✓				✓												✓
Hoffmann et al., 2009 (29)	12	12 (same patients)	Depression										✓		✓					
Lang et al., 2010 (30)	7	7	Schizophrenia										✓		✓					
Majic et al., 2013 (31)	27	27	Dementia					✓						✓	✓					
Nathans-Barel et al., 2005 (32)	10	10	Schizophrenia		✓	✓									✓					
Nordgren et al., 2014 (33)	20	13	Dementia	✓								✓			✓					
Nurenberg et al., 2015 (34)	49	41	Schizophrenia or admission due to forensic reasons											✓	✓				✓	
Schramm et al., 2015 (15)	6	0	Depression				✓	✓	✓							✓				
Sockalingam et al., 2008 (35)	1	0	Bipolar disorder, currently depressive after an assault	✓		✓		✓		✓	✓				✓					

Overview of the included studies showing population details, examined variables, and type of AAT. AAT, animal-assisted therapy. The checkmarks indicate which variables were examined and which type of AAT was used. Blue shading refers to reduced symptomatology in the AAT-intervention group as compared to the control group or over the course of time, while yellow shading refers to aggravated symptomatology after an AAT-intervention.

### Effects of AAT on Anhedonia and Quality of Life

Nathans-Barel et al. (32) reported positive effects of a dog-assisted intervention compared to a psychosocial treatment in persons suffering from chronic schizophrenia. Furthermore, they reported a positive effect on their quality of life.

### Effects of AAT on Mindfulness, Depression, and Rumination

Schramm et al. (15) examined the effect of a sheep-assisted therapy in depressive patients within the framework of a mindfulness based approach. The intervention was practicable and led to reduced depressive symptoms and rumination, while the ability for mindfulness was increased.

Sockalingam et al. (35) report the case of a patient who, following an assault with a concurrent mood disorder, profited greatly from a dog-assisted intervention over a 3-week period.

### Effects of AAT on Self-Efficacy and the Ability to Cope

Berget et al. (28) conducted 12 weeks of AAT in persons with schizophrenia, affective disorders and personality disorders. They found a significant improvement in self-efficacy and the ability to cope, but no difference in general quality of life.

### Effects of AAT in Patients With Dementia

Peluso et al. (36) examined AAT in patients with dementia and found positive influence on anxiety and aggressiveness. Nordgren and Engstrom (33) observed positive effects of a dog-assisted intervention on behavioral symptoms in dementia. Majic et al. (31) examined the influence of a dog-assisted therapy on agitation/aggression and depression in persons with dementia. They found constant frequency and severity of symptoms of agitation/aggression and depression in the group with AAT while the control group not receiving AAT displayed a significant increase of the symptoms.

### Effects of AAT on Anxiety

Lang et al. (30) examined symptoms of anxiety before and after a dog-assisted therapeutic session in persons with acute psychotic symptoms. Also, they observed a significant reduction of anxiety and fear after the session. In depressive patients, Hoffmann et al. (28) observed that a single therapeutic session with a dog led to reduced fear as opposed to a session without a dog. Furthermore, Barker and Dawson (26) compared the effects of an AAT session with those of a regularly scheduled therapeutic recreation session using a pre- and posttreatment crossover study design in 230 patients. They found reductions in anxiety scores after the AAT session for patients with psychotic disorders, mood disorders, and other disorders, and after the therapeutic recreation session for patients with mood disorders. However, there were no significant differences in reduction of anxiety between the two types of sessions (26).

### Effects of AAT on Aggression

Nurenberg et al. (34) examined the effect of horse- or dog-assisted therapy in chronically ill psychiatric patients with a history of violent behavior (at least three committed violent acts in the last 12 months). They observed that both AAT interventions reduced aggressiveness in these patients.

### Benefit-Risk-Assessment

In addition to the expected benefits of AAT, close contact of animals and humans always bears risks, such as allergies, infections, and animal-related accidents (37). A systematic review by Bert et al. (37) including 36 studies on children, psychiatric, and elderly patients shows that the benefits of AAT greatly outweigh its risks. Furthermore, the authors suggest that the implementation of simple hygiene protocols is sufficient to minimize the risk of infections.

## Discussion

The question whether AAT could help to prevent aggression and coercion in psychiatry has not been specifically addressed in a review thus far. A number of studies have examined the effects of AAT in psychiatric patient samples, and some authors have tried to summarize the results of these studies with different research objectives in systematic and non-systematic reviews. We found studies reporting positive effects of AAT on quality of life, mindfulness, depression, rumination, self-efficacy, dementia, anxiety, and aggression. The results are in line with the previous studies stating the various positive effects of AAT in different mental health settings. Still, qualitative and quantitative syntheses are complicated due to small sample sizes and methodological limitations of the published studies. In particular, there is limited evidence for the specificity of many positive findings in AAT, necessitating future research with more advanced study protocols.

However, in the current narrative expert review including a systematic literature search, we found non-systematic indications of a positive effect of AAT on different psychiatric conditions connected with risk behavior and coercion, in particular with anxiety and aggression. Furthermore, there is a broad consensus that the benefits of AAT greatly outweigh its risks.

### Strengths

We examined AAT from a new perspective taking into account its potential implications for the reduction of coercive treatment. Due to this strong clinical implication, we consider it a highly relevant topic. Furthermore, our literature search is up to date and systematic.

### Limitations

Multiple factors constitute methodological limitations of this review. Due to the current state of the literature, a narrative expert review was conducted, and some publications on the subject could have been overlooked. Also, the literature search, selection process, and data extraction have been conducted by one single author. Furthermore, no systematic quality assessment of the included publications was performed, and an analysis of publication bias and quantitative synthesis of the findings were not possible. However, this approach is adequate considering the current state of the field. Further limitations of this study are that, although we found indications that AAT may help to reduce coercive measures in psychiatry, we did not elaborate a concept on how to implicate AAT in acute psychiatric settings. Also, we found no study examining directly the influence of AAT on coercion in psychiatry. Future research may show if the current non-systematic evidence for a potential use of AAT can be replicated and corroborated.

### Clinical Implications

**Figure 2:**
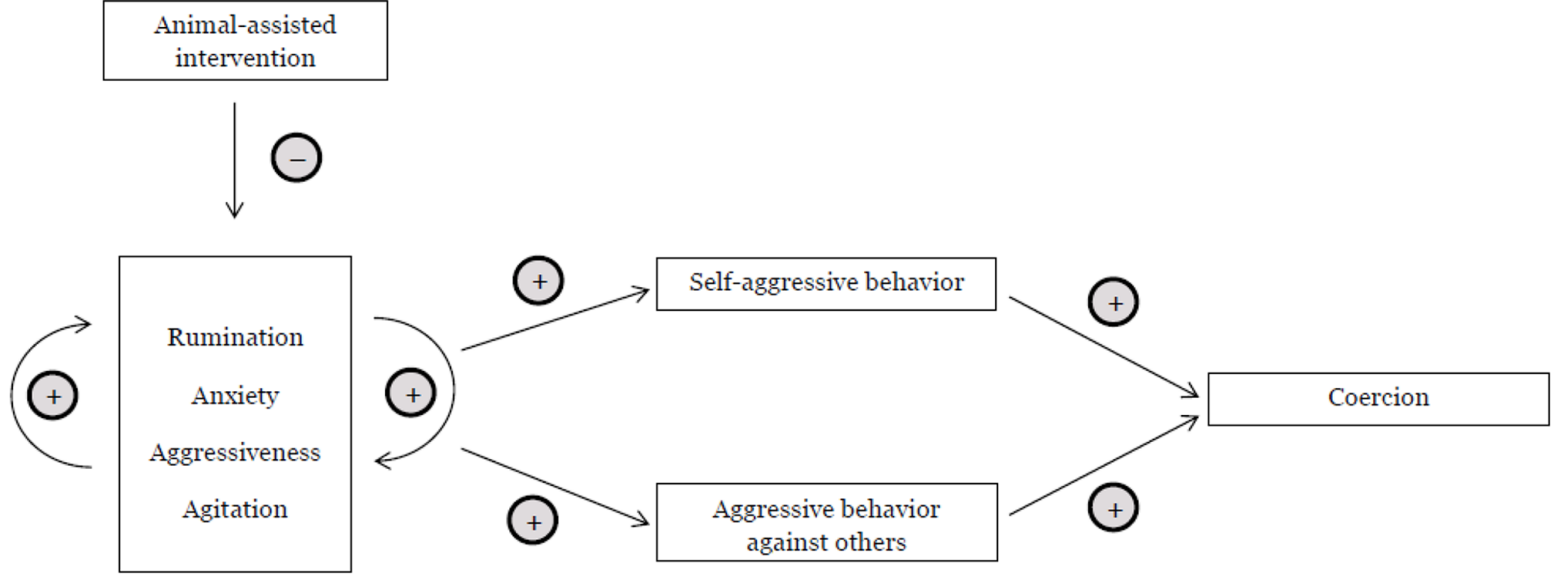
Deescalative Potential of AAT. This figure shows potential connections between psychiatric sypmtom domains, aggressive behavior, and coercion. –: Increase (decrease) in the previous domain leads to a decrease (increase) in the following domain. +: Increase (decrease) in the previous domain leads to an increase (decrease) in the following domain.

AAT greatly benefits human health—it enhances our general wellbeing as well as it enables persons with psychiatric diseases to achieve better therapeutic outcomes in many different areas and for a variety of symptoms. In the context of coercive treatment in psychiatry, we highlight the promising potential of AAT to relieve symptoms leading to aggressive behavior. We hypothesize that applying AAT in psychiatric wards reduces the need for coercive treatment. Furthermore, we think that even at later stages in the escalation process leading to a coercive measure, AAT could deescalate the situation so far as to render the coercion unnecessary. It has been shown that aggressiveness itself diminishes in the presence of a therapeutic animal. **Figure 2** shows the process in which AAT could deescalate and prevent coercive measures.

We suggest implementing AAT as a low-threshold therapeutic measure in psychiatric wards as a social psychiatric mean to minimize coercion. We hypothesize that the presence of an animal at, for example, the department for persons suffering from schizophrenia would lead to a decline of agitation in the patients of the ward—this could improve the general atmosphere of a ward. Furthermore, we hypothesize that, if a certain therapy animal is known to the patients, this animal could calm persons even in high-risk situations and subsequently enable mutual agreement between the patient and the therapist, which in turn would allow for a better relationship between patient and animal and lead to an enhanced treatment compliance with less and less coercive treatment necessary.

## Conclusion

Based on our findings, we suggest integrating AAT into an aggression-reducing setting, potentially combining it with an enriched environment, music therapy, and other supportive therapies in addition to established psychotherapy and psychopharmacotherapy. AAT could thus be implemented as one of multiple non-pharmacological treatment approaches. Systematic studies, however, are needed to confirm the hypothesis that AAT can help to prevent coercive treatment.

## Author Contributions

SW and CH designed the study and wrote the initial draft of the paper. SB and UL revised the manuscript for important intellectual content. All authors have contributed to, read, and approved the final version of the manuscript.

## Conflict of Interest

The authors declare that the research was conducted in the absence of any commercial or financial relationships that could be construed as a potential conflict of interest.
